# The Association Between Sedentary Screen Time, Non-screen-based Sedentary Time, and Overweight in Chinese Preschool Children: A Cross-Sectional Study

**DOI:** 10.3389/fped.2021.767608

**Published:** 2021-12-23

**Authors:** Rui Hu, Hui Zheng, Congchao Lu

**Affiliations:** ^1^Department of Endocrinology, TEDA International Cardiovascular Hospital, Tianjin, China; ^2^School of Public Health, Tianjin Medical University, Tianjin, China; ^3^Tianjin Key Laboratory of Environment, Nutrition and Public Health, Tianjin, China

**Keywords:** media exposure, sedentary screen time, non-screen-based sedentary time, overweight and obesity, preschool children

## Abstract

**Introduction:** Less is known about the effects of the different domains of sedentary behaviors on healthy weight in young children. This cross-sectional study examined the association between sedentary screen time (SST), non-screen-based sedentary time (NSST), and overweight (and obesity) in Chinese preschoolers.

**Methods:** Data were collected from the Physical Activity and Health in Tianjin Chinese Children study (PATH-CC), involving healthy children 3–6 years old and their families. Children's overweight status was classified according to the international (IOTF) childhood BMI cut-offs. SST and NSST were reported in minutes/day by parents using the leisure-time sedentary behaviors questionnaire. Logistic regression models adjusted by sex, age, socioeconomic status, outdoor play, and sleep duration were used.

**Results:** In a total of 971 children (55.4% boys), 11.8% were overweight. Generally, children spent 1 h/day in SST and 1 h/day in NSST. Multiple models showed that children who spent more time in SST were more likely to be overweight [OR and 95% CI: 1.22 (1.03–1.45)]. No correlation between time spent on NSST and children with overweight was found (*P* > 0.05).

**Conclusions:** This study indicated that children who spent more time in SST were more likely to be overweight, but a null correlation between NSST and overweight was found. Longitudinal studies designed to identify associations between exposures to screen media and changes in metabolic parameters during a child's early years are needed.

## Introduction

The increasing prevalence of childhood overweight (and obesity) has become one of the most critical threats to public health in all countries. Globally, the prevalence of overweight among children and adolescents aged 5–19 years has risen dramatically from 4% in 1975 to over 18% in 2016, and 39 million children under five were overweight or obese in 2020 (1). In low- and middle-income countries, people are also experiencing a rapid rise in obesity. For example, in China, the prevalence of overweight in children and adolescents has increased from 6.7% in 1991–1995 to 18.5% in 2011–2015 (2). Children with obesity are at greater risk of obesity later in life, e.g., a systematic review summarized that children and adolescents with obesity were around five times more likely to be obese in adulthood than individuals without obesity (3). In addition, childhood overweight is associated with type 2 diabetes and cardiovascular disease in later life. Previously, school-based health lifestyle intervention in China yielded a slight to null effect on obesity in older children (aged 6–18 years). Thus, obesity intervention in childhood is needed as early as possible (4). The preschool years stand for a critical period for children to develop individual life behaviors and may produce more effective effects on maintaining healthy body weight in children. Evidence suggests that early and effective prevention may be the key to controlling the global obesity epidemic (5). Therefore, recognizing the determinants of overweight during the preschool years is essential for designing a lifelong weight management plan (6).

It is well known that body weight gain is related to unhealthy lifestyles, such as increased energy intake caused by high-density energy foods and reduced activity energy expenditure caused by insufficient physical activity. In the last decade, people are paying more and more attention to the correlation between sedentary behavior (SB) and the high levels of adiposity (7). Emerging evidence shows that the home media environment was most consistently associated with adiposity in childhood (8). The World Health Organization (WHO) recommended that children limit the amount of time spent being sedentary, particularly recreational screen time (9). While a systematic review summarized that there was no association between accelerometer-derived SB and adiposity among preschool children (10), perhaps the relationship between screen-based SB and overweight cannot be fully explained from the perspective of total activity energy expenditure reduction alone. In addition, it should be noticed that different domains of SB may have different health effects. For example, non-screen-based SB such as reading or studying would be favorable for cognitive development in children. However, there is a lack of evidence available to describe the associations between the various domains of SB and healthy weight in young children.

Identifying the associations between SB and children with overweight is essential for informing the public which domain of SB should be a potential intervention target for healthy weight (11). Leisure time at preschool age has the potential to make a substantial contribution to a child's daily SB level. For example, in Chinese preschool children, less active children spent a large proportion of time sedentary during after-school hours on school days and the weekends/holidays (12). This cross-sectional study aimed to examine the association between leisure-time sedentary screen time (SST), non-screen-based sedentary time (NSST), and overweight in Chinese preschool children. It was hypothesized that children who spend more time in SB would be more likely to be overweight than those who do not, and this correlation might depend on whether the results were screen-based or not. Such evidence would better understand the modality (screen-based SB vs. non-screen-based SB) with health indicators among young children in modern urban cities in China.

## Methods

### Data Collection

Data were derived from the Physical Activity and Health in Tianjin Chinese Children study (PATH-CC), which focused on identifying the relationship between environmental determinants, physical activity, and overweight among children in Tianjin, China. Details of the study have been reported elsewhere (12, 13). In total, 1,031 healthy children and their parents participated in the study, and the participation rate was 93.7% (i.e., 1,031 out of 1,100 children in the recruited preschools). Child anthropometry measurements and questionnaire data were collected in May 2015. Children's height and weight were measured by trained school nurses in preschools, and children with overweight (and obesity) were classified according to the international (IOTF) childhood BMI cut-offs (14). Questionnaires were sent to parents for data collection, including child and family information, child SST and NSST during leisure time, and outdoor play. Five children missed the anthropometry measurements during the data collection, and 55 children had no household income or sleep information. Missing data (5.8%) were not imputed. Written informed consent was obtained from the parents. This study was approved by the Medical Ethics Committee of the Tianjin Medical University (TMUhMEC2014001) and performed following the Declaration of Helsinki.

### Leisure-Time Sedentary Behaviors Questionnaire

Parents reported children's SST and NSST during leisure time *via* the leisure-time sedentary behaviors questionnaire (LTSB_q_). The LTSB_q_ was a valid questionnaire with an acceptable reliability that compared to the results of ActiGraph (ρ = 0.365, *P* = 0.019) (13). Leisure time was defined as the time spent outside of school hours on weekdays (including interest classes after school and time spent outdoors and at home, but not travel time) and entire weekend days. SST includes watching TV; playing with a mobile phone, tablet, or electronic games; and using a computer in this study. NSST includes reading, writing, or drawing and playing with toys (stationary but moving arms). Parents reported the frequency and duration of these behaviors. For example, the questionnaire asked how many minutes per day, on average, their children spent on each item during leisure time on schooldays in the past week (response categories ranged from 0 to 5 days) and on days over the past weekend (response categories ranged from 0 to 2 days). The average number of minutes was computed to obtain an overall average SST or NSST per day.

### Potential Correlates

Parents reported child outdoor play and overnight sleep duration (bedtimes and wake times) in the past 7 days, and both were separate for school and weekend days (15, 16). Outdoor play was reported in terms of frequency and duration. Outdoor play and sleep durations were given in the mean of hours per day. Childhood socioeconomic status (SES) was categorized into low SES (< RMB 30,000/person/year), middle SES (RMB 30,000–50,000/person/year), and high SES (>RMB 50,000/person/year) based on parental-reported household income.

### Statistical Analysis

Continuous variables were presented as means with SDs, or, if data were skewed, as the median with 25th−75th percentile. Categorical variables were presented as rates in number and percentages. Differences in categorical variables were tested by χ^2^ test. Correlations between SST, NSST, outdoor play, and sleep duration were checked by Spearman correlation since data were skewed. Logistic regression models were used to determine the relationships between SST, NSST, and children with overweight. Models were adjusted for child age, sex, socioeconomic status, outdoor play, and sleep time. IBM SPSS Statistics V.26 for Windows was used for this study, with test level α = 0.05, and analyses were conducted in 2021.

## Results

**Figure 1:**
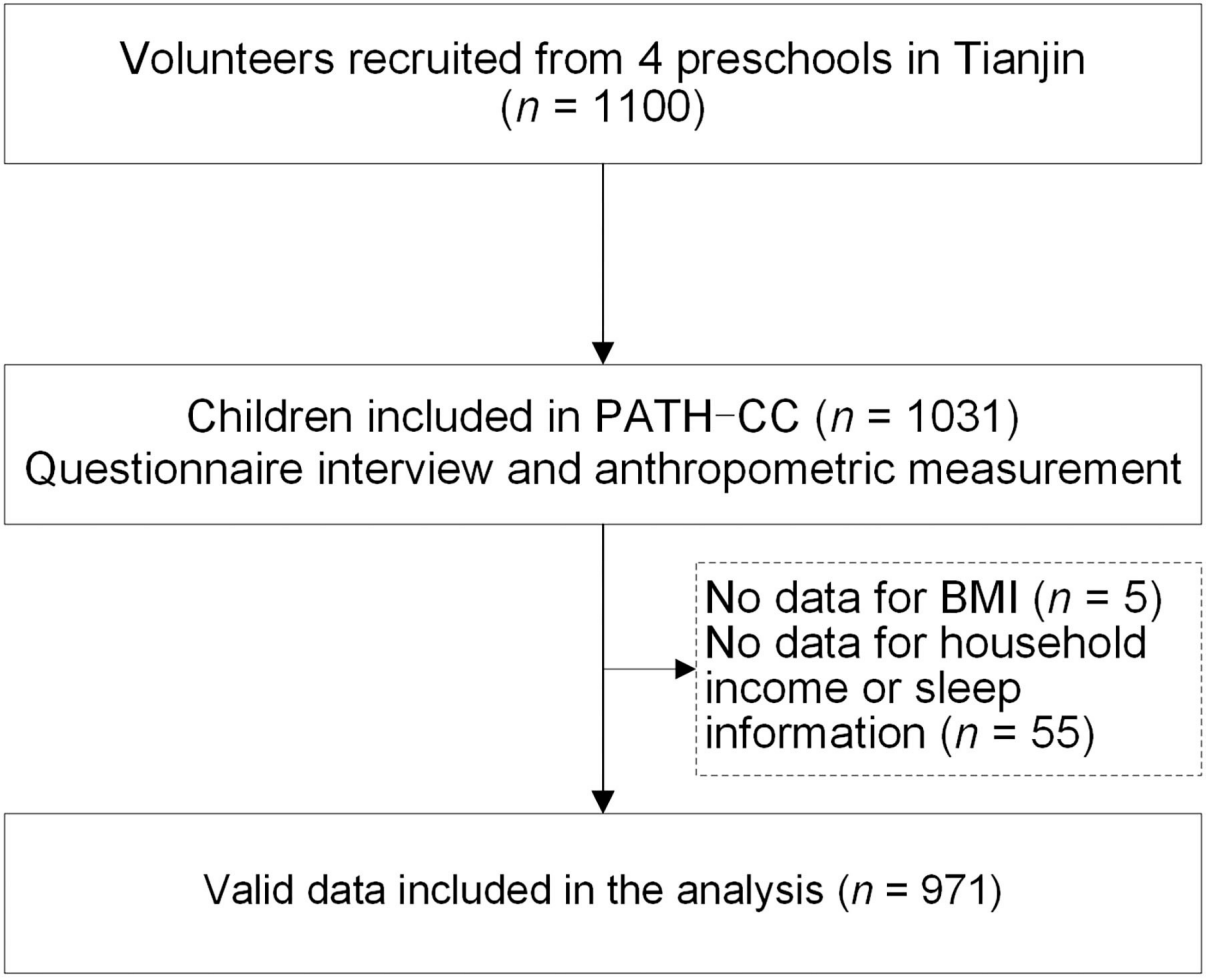
Flowchart of participants in the PATH-CC study.

The characteristics of the study population are presented in Table 1. A total of 971 children (55.4% boys), aged 4.8 ± 1.1 years, had valid data and were included in this analysis (Figure 1). There were 11.8% of those children with overweight or obesity. During leisure time, children spent a median of 1 h/day on SST [60 (31–07) min/day], and more than half of the children (*n* = 519, 53.5%) spent more than 1 h/day. The average level of NSST [60 (36–90) min/day] was similar to the time spent on SST, and the median was also 1 h/day. In general, children spent 26 (17–45) min/day of outdoor play and 9.5 (9.0–9.9) h/day of sleep overnight. In addition, more SST was associated with less outdoor play (ρ = −0.081, *P* = 0.011) and less sleep duration (ρ = −0.116, *P* = 0.000), and no correlation between NSST and outdoor play or sleep time (all *P* > 0.05) was found in this study.

**Table 1:** Characteristics of the study population in the PATH-CC study (*n* = 971).

**Variable**	**Range**	**Non-overweight** **(*n* = 856)**	**Overweight and obese** **(*n* = 115)**
Sex	Male	459 (53.6%)	79 (68.7%)
	Female	397 (46.4%)	36 (31.3%)
Age	3–6 (years)	4.7 ± 1.1	5.6 ± 0.7
Body mass index	11.9–25.7	15.1 ± 1.1	19.0 (17.7; 20.1)
Ethnicity	Han nationality	805 (94.0%)	107 (93.0%)
	Other nationalities	51 (6.0%)	8 (7.0%)
Socioeconomic status^*^	Low	218 (25.5%)	47 (40.9%)
	Middle	271 (31.7%)	38 (33.0%)
	High	367 (42.9%)	30 (26.1%)
Outdoor play	0–2.5 (h/day)	0.4 (0.3; 0.8)	0.5 (0.2; 0.8)
Sleep duration	7–11.6 (h/day)	9.5 ± 0.6	9.3 ± 0.7
Sedentary screen time	0–8 (h/day)	1.0 (0.5; 1.7)	1.3 (0.7; 2.1)
Non-screen-based sedentary time	0–8.4 (h/day)	1.0 (0.6; 1.5)	1.1 (0.6; 1.6)

* *Childhood socioeconomic status was categorized into low SES (< RMB 30,000/person/year), middle SES (RMB 30,000–50,000/person/year), and high SES (>RMB 50,000/person/year) based on household income*.

In Table 2, the multiple logistic regression results showed that SST was correlated with children with overweight. It indicated that those children who spent an additional hour more SST a day were almost 1.22 [95% CI: (1.03–1.45)] times more likely to be overweight. However, there was no correlation between time spent on NSST and children with overweight (*P* > 0.05). Results showed that boys and older children had a higher proportion of overweight, and children from high SES families were more likely to be overweight than the middle groups.

**Table 2:** The association between screen time and children with overweight and obesity by logistic regression analysis among preschoolers in the PATH-CC study (*n* = 971).

**Correlates**	**Code/range**	**OR (95% CI)**
Sex	0 = female; 1 = male	**1.63 (1.05; 2.54)**
Age (years)	3–6	**2.42 (1.90; 3.08)**
Socioeconomic status (SES)	Middle SES as reference	
	Low SES	1.36 (0.83; 2.24)
	High SES	**0.57 (0.33; 0.97)**
Outdoor play (h/day)	0–2.5	1.46 (0.94; 2.29)
Sleep duration (h/day)	7–11.6	0.78 (0.54; 1.12)
Sedentary screen time (h/day)	0–8	**1.22 (1.03; 1.45)**
Non-screen-based sedentary time (h/day)	0–8.4	1.13 (0.90; 1.42)

*Bold: P < 0.05*.

## Discussion

This study reports correlations between screen-based SB, non-screen-based SB, and overweight in Chinese preschool children. These results showed that children who spent more time on SST were more likely to be overweight. However, no correlation between NSST and children with overweight was found. These findings highlight the importance of reducing children's screen-based SB to control or prevent the development of overweight and obesity in preschool years.

The youth's excessive sedentary behavior has become a growing public health concern, and there has been progressive growth in research studying SB and health effects in children and adolescents. Based on the literature, SB is typically defined as any waking behavior characterized by an energy expenditure ≤ 1.5 metabolic equivalents (METs) while in a sitting or reclining posture (17). This study examined two sorts of SB and their correlation with childhood overweight, and different results were found. That is, in preschool years, screen-based SB may have an unfavourable effect on healthy weight, while non-screen-based SB is likely to have a null effect on healthy weight (18). Over the past century, people have gotten used to screen-based equipment (e.g., television, computers, mobile phones, etc.) for working or studying and entertainment during leisure time. Previously, some researchers assumed the association between screen time and childhood overweight as children might also be exposed to more food advertisements while on their screens, thus influencing their eating behaviors, e.g., increased eating while viewing and caused more calorie intake. The latest evidence showed that the increasing screen exposure made people shift away from nature's 24-h day/night rhythm and thus may cause disruptions in sleep patterns and altered circadian clock function (19, 20). Recent studies have indicated that high artificial light levels, either outdoor or in the bedroom, correlate with increased body weight in adults. This fact might be explained by the exposure to light of shorter wavelengths associated with altered sleep–wake cycle, daily rhythms of food intake, and disturbed metabolism (21, 22). However, it is not clear what are the metabolic consequences of chronic screen light exposures in young children. Given the increased incidence of type 2 diabetes and cardiovascular disease at a younger age, longitudinal studies designed to identify the associations between exposures to screen media and changes in metabolic consequences over time and to generate hypotheses about causal relationships are needed (23).

Healthy sleep is an essential component of child growth and development. A systematic review summarized that shorter sleep duration was associated with higher adiposity in children aged 0–4 years (24). In this study, child sleep duration was considered a potential correlate, but a null association was found. Screen use was found to be associated with shortened sleep duration in adults (25). This study also found that longer screen time was associated with shorter sleep duration (ρ = −0.116, *P* = 0.000). The WHO strongly recommended that preschool children (3–4 years old) reduce daily sedentary screen time to <1 h and have 10–13 h of good-quality sleep with regular sleep and wake-up times for better health benefits (26). In this study, the median of overnight sleep duration was 9.5 (9.0; 9.9) h/day, and more than half of the children (*n* = 519, 53.5%) could not meet this screen time recommendation. Perhaps lower unessential screen time could also benefit from promoting an adequate time for overnight sleeping in children. In addition, except for adequate sleep duration, healthy sleep also includes good quality, the absence of sleep disturbances or disorders, and appropriate timing. For example, one study reported that later bedtime was associated with higher adiposity indices in early adolescents from southern China (27). While there are less evidence about other important sleep indicators on heathy weight beyond sleep duration at the moment, it should be noticed that light plays a critical role in modulating sleep patterns, and whether screen light exposure before bedtime in early childhood could disturb current sleep patterns and continue to cause a long-term sleep pattern change needs further evaluation (28).

Over the past decade, there have been considerable changes in children's use of screens. For example, the new digital media are increasingly pervasive in the lives of children in the household, e.g., mobile phones and video content online. A systematic review summarized that children with greater access to screen media devices at home were more likely to have higher use of those devices (29). During the COVID-19 pandemic, a daunting increase in leisure screen time consumption in children and adolescents was reported (30). Take an example of China, a survey of school children and adolescents (*n* = 2,426, 5–17 years old) reported an approximately 4 h/day increase in leisure screen time when evaluated before (January 2020) and after (March 2021) the COVID-19 pandemic lockdown (31). It is well known that parental factors such as their rules on child screen time (32) or parental SES characteristics played an essential role in limiting the child's screen time in preschool years. For example, previous studies reported that higher parental educational levels were inversely associated with high screen time in children under 3 years of age (33). Interestingly, one study indicated that more educated parents provide more opportunities for their children to engage in screen-based behaviors while working from home during the pandemic (34). It should be noticed that using a single SES indicator (e.g., parental education or household income) would not capture all socioeconomic position dimensions of a family. Therefore, a standardized and synthesized SES indicator is needed for harmonized comparable socioeconomic position across populations (35). Lifestyle behaviors established early in life tend to track over time, e.g., childhood TV viewing time has been reported to track into adulthood (36). The preschool years are seen as a critical window for predicting childhood weight gain. Thus, future studies need to identify the barriers and facilitators that can help to lower child screen time in different socioeconomic groups in the early years.

Further research exploring the correlation between SB and health indicators is also needed to clarify their definitions (37) to better understand the role of SB modality and children's development. Some unclear definition of SB can easily lead to confusion, for example, a mixed use of “sedentary behavior” and “screen time,” which cannot be interchanged in health-related research, and incorrect conclusions may derive (38). It should be noticed that children's large amounts of SB are not necessarily due to high media use, e.g., a study in Germany reported that primary school children spent 81 min daily using screen media, which corresponds to almost half (46.2%) of their total sedentary time (39).

To the best of our knowledge, this is the first study that reports the association between SST, NSST, and children with overweight in the same group of Chinese preschoolers. Such evidence could support a better understanding of different domains of SB concerning healthy weight in young children. Measurements of SST and NSST were based on a valid questionnaire with an acceptable quality that was validated using accelerometry. Limitations of the present study include its cross-sectional design, so findings must be interpreted with caution. Additionally, children's diet factor was not assessed in this study, but it could have an effect on their weight status and needs to be studied further. Children in this study were recruited as volunteers and derived from a random sample in one of the most important urbanized cities in China. Therefore, our hypotheses need to be tested further in a larger sample from the representative socioeconomic populations; furthermore, it is unknown how these results would translate to other nations since different cultures affect different cultural habits in children's SB patterns.

## Conclusions

This study examined the impact of screen-based SB compared with non-screen-based SB on children with overweight; sleep duration and outdoor play were considered confounders. The results showed that children who spent more time in SST were more likely to be overweight, but a null correlation between NSST and overweight was found. These findings implicate the importance of reducing screen time in children to control or prevent the development of overweight and obesity in preschool years. Furthermore, this study indicated that leisure time could be a potential period for early life obesity interventions during childhood. There is a need to investigate the association between device-based measures of screen-based SB and metabolic parameters in preschool years, with a longitudinal study design to test the influence of long-term changes.

## Data Availability Statement

The datasets used and analysed during the current study are available from the corresponding author on reasonable request.

## Ethics Statement

The study was approved by the Medical Ethics Committee of the Tianjin Medical University (TMUhMEC2014001) and performed following the Declaration of Helsinki. Written informed parental consent was obtained for participation in the study.

## Author Contributions

CL and RH designed the study. RH, HZ, and CL were involved in the data analysis and interpretation. RH drafted the manuscript. All authors contributed to the article and approved the submitted version.

## Funding

This study was supported by TEDA International Cardiovascular Hospital (2021-TD-009), the Health Commission of Tianjin Binhai New District (2016BWKY020), and Tianjin Medical University.

## Conflict of Interest

The authors declare that the research was conducted in the absence of any commercial or financial relationships that could be construed as a potential conflict of interest.

## Publisher's Note

All claims expressed in this article are solely those of the authors and do not necessarily represent those of their affiliated organizations, or those of the publisher, the editors and the reviewers. Any product that may be evaluated in this article, or claim that may be made by its manufacturer, is not guaranteed or endorsed by the publisher.
